# Improvement of Occlusal Function After Clear Aligner Orthodontics Verified by T-Scan Novus Digital Analysis

**DOI:** 10.3390/reports9010058

**Published:** 2026-02-11

**Authors:** Tanya Bozhkova, Nina Musurlieva, Velina Stoeva

**Affiliations:** 1 Department of Prosthetic Dentistry, Faculty of Dental Medicine, Medical University of Plovdiv, 4002 Plovdiv, Bulgaria; 2 Department of Social Medicine and Public Health, Faculty of Public Health, Medical University, 4000 Plovdiv, Bulgaria; 3 Department of Epidemiology and Disaster Medicine, Faculty of Public Health, Medical University, 4000 Plovdiv, Bulgaria; velina.stoeva@mu-plovdiv.bg

**Keywords:** digital occlusal analysis, T-Scan Novus, clear aligners, occlusal force distribution, case report

## Abstract

**Background and Clinical Significance**: Clear aligner therapy has become a widely used orthodontic treatment, particularly among adults seeking esthetic and comfortable alternatives to fixed appliances. Achieving a stable and functional occlusion remains one of the primary objectives in orthodontics. The T-Scan digital occlusal analysis system offers an innovative and objective method for quantifying occlusal contact distribution and timing, thereby improving diagnostic accuracy and follow-up. This report aims to present a clinical case demonstrating the use of the T-Scan Novus system for evaluating occlusal balance before and after clear aligner therapy, highlighting its role in documenting short-term functional occlusal changes. **Case presentation**: A 42-year-old female patient with Class II malocclusion, deep bite, and anterior crowding was treated with Smilers^®^ clear aligners over nine months (18 aligners). Digital occlusal analysis was performed before treatment and one month after treatment. Pre-treatment analysis demonstrated a pronounced asymmetry in occlusal force distribution, with left-side dominance (67.9%) compared with the right side (32.1%). One month after treatment, occlusal forces were more evenly distributed (52.4% left, 47.6% right). Occlusion time decreased to 0.25 s and disocclusion time to 0.08 s, falling within commonly reported physiological ranges. **Conclusions**: Within the limitations of a single-case design and short-term follow-up, digital occlusal analysis using the T-Scan Novus system enabled objective documentation of occlusal force distribution and timing changes after clear aligner therapy. These findings are descriptive and hypothesis-generating and should be interpreted cautiously.

## 1. Introduction and Clinical Significance

The demand for adult orthodontic treatment has steadily increased, driven by a preference for esthetic, comfortable, and minimally invasive treatment modalities [1,2]. Clear aligner therapy (CAT) has therefore become a widely accepted alternative to fixed appliances, offering advantages such as improved oral hygiene, enhanced patient comfort, and reduced visibility [3,4].

An additional benefit of aligner therapy is the avoidance of metallic components, which may be particularly relevant for patients with known or suspected metal hypersensitivity [5]. Thermoplastic aligner materials are generally considered biocompatible and well tolerated intraorally [6]. Nevertheless, the increasing global use of aligners has raised concerns regarding environmental sustainability, as the sequential manufacturing and disposal of multiple aligners per patient contribute to plastic waste [7].

Beyond esthetic alignment, the primary objective of orthodontic treatment is the establishment of a stable and functional occlusion [8]. Asymmetric occlusal force distribution and prolonged contact timing may compromise masticatory efficiency and long-term occlusal stability, even in patients without overt temporomandibular symptoms [9]. Conventional occlusal indicators, such as articulating paper, provide primarily qualitative information and lack objective quantification of force magnitude and timing [10].

Digital occlusal analysis using the T-Scan Novus system enables dynamic and reproducible assessment of occlusal force distribution and contact timing [11,12]. Although previous studies have investigated occlusal parameters following orthodontic treatment at a population level [13,14], clinical illustrations focusing on digital occlusal assessment after clear aligner therapy remain limited.

The novelty of this case report lies in the clinical illustration of digital occlusal analysis using the T-Scan Novus system to objectively document short-term changes in occlusal force distribution and contact timing following clear aligner therapy.

### Aim of the Study

This report aims to present a clinical case demonstrating the use of the T-Scan Novus system for evaluating occlusal balance before and after clear aligner therapy, highlighting its contribution to documenting short-term functional occlusal changes.

## 2. Case Presentation

### 2.1. Diagnosis and Etiology

A 42-year-old female patient presented with esthetic concerns related to anterior dental misalignment. Clinical and radiographic examination revealed a Class II malocclusion with retroclination of the maxillary central incisors, increased overbite (deep bite), and mild anterior crowding. No clinically relevant transverse discrepancies or midline deviations were observed. Periodontal tissues were clinically healthy at baseline, and the patient reported no temporomandibular joint pain or masticatory discomfort.

**Figure 1:**
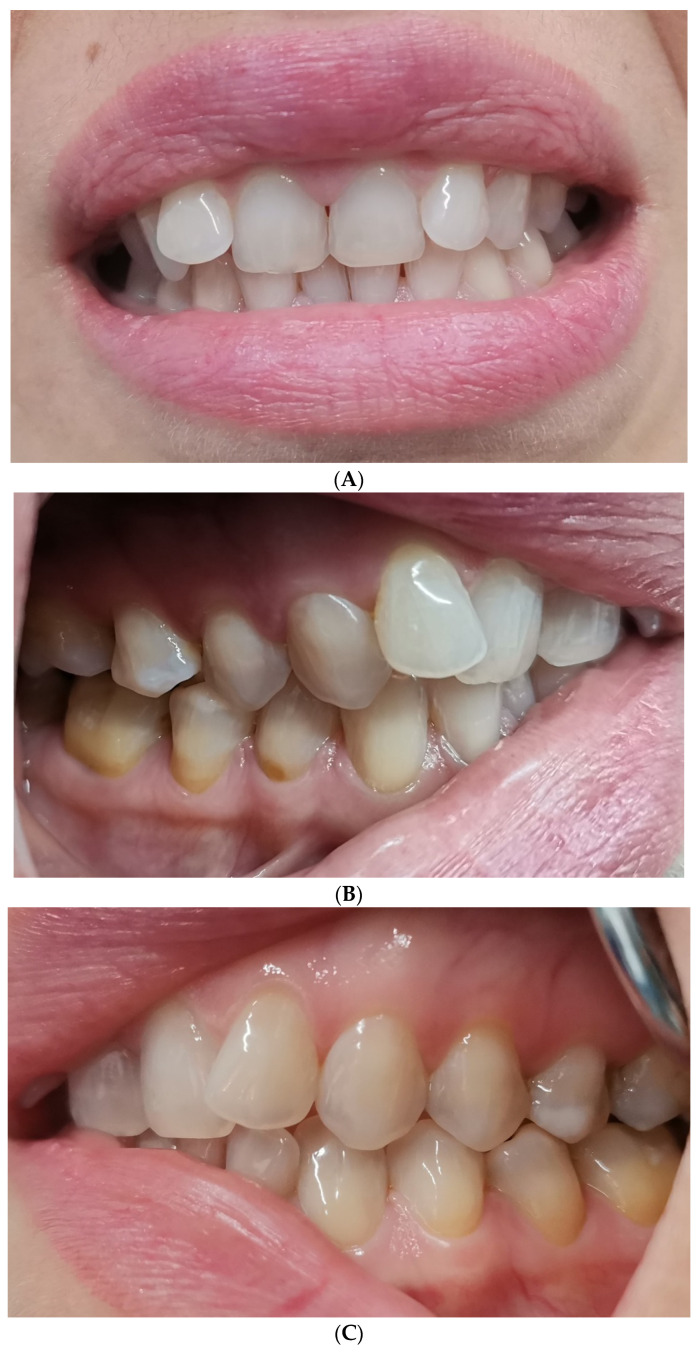
(**A**–**C**) Intraoral photos in maximum intercuspation position (MIP).

**Figure 2:**
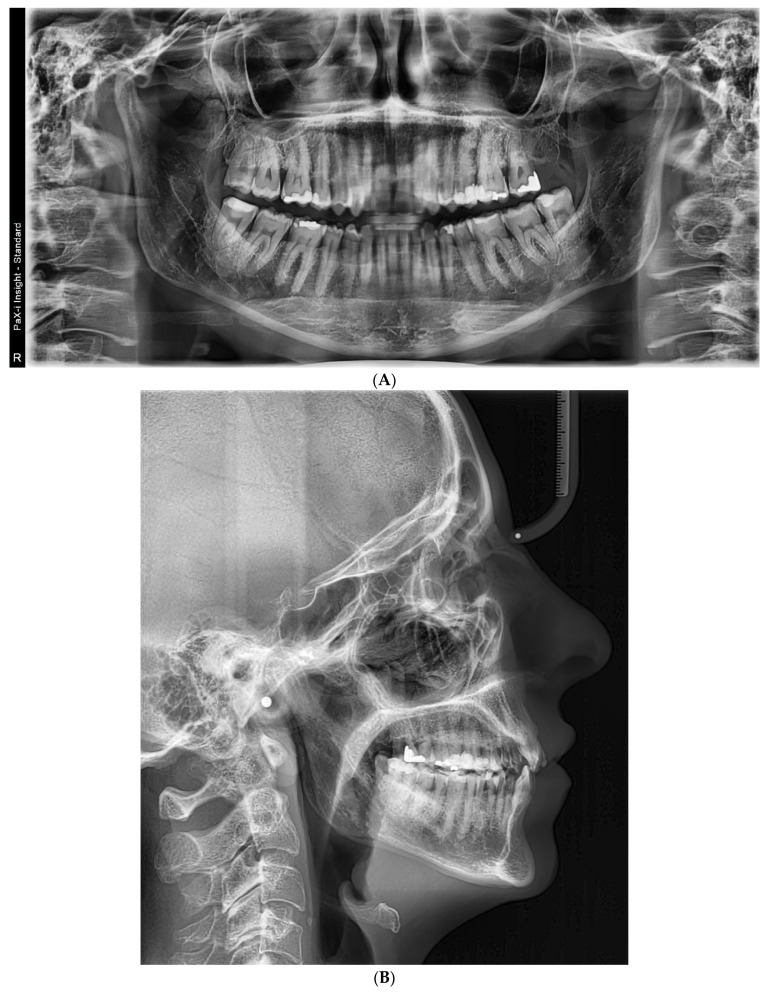
(**A**) Initial panoramic radiograph; (**B**) lateral cephalogram.

Baseline intraoral photographs and radiographic findings are presented in Figure 1 and Figure 2.

### 2.2. Treatment Progress

The patient declined fixed orthodontic treatment and opted for clear aligner therapy. Intraoral scans were obtained using a Medit i500 scanner and submitted to Smilers^®^ (Biotech Dental, Salon-de-Provence, FranceB, France) for digital treatment planning.

**Figure 3:**
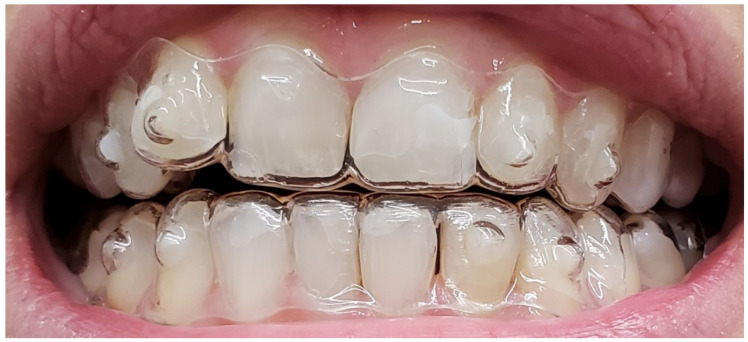
Smilers^®^ clear aligners, Biotech Dental, France.

The treatment plan included limited interproximal enamel reduction in the anterior region to relieve crowding and facilitate alignment. A total of 18 sequential aligners were prescribed and changed every two weeks, resulting in a total treatment duration of nine months. Attachments were placed according to the digital treatment plan. No intermaxillary elastics or post-treatment occlusal adjustments were performed prior to the post-treatment occlusal analysis. Figure 3 illustrates the aligners following completion of the initially planned interproximal reduction.

### 2.3. Digital Occlusal Analysis

**Figure 4:**
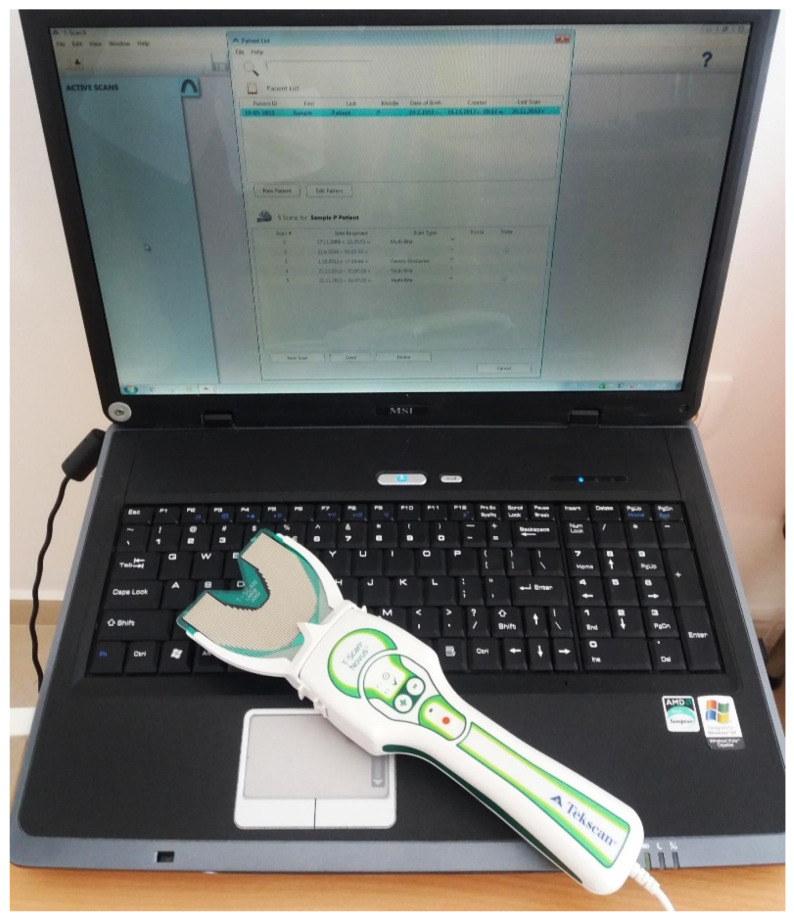
T-Scan Novus system setup.

Digital occlusal analysis was performed using the T-Scan Novus System (Tekscan Inc., South Boston, MA, USA) (Figure 4) with a Novus HD sensor. Prior to each recording session, the system was calibrated according to the manufacturer’s instructions.

All recordings were performed by the same experienced operator. The patient was seated upright with standardized instructions to close into maximum intercuspation with comfortable force. Three consecutive recordings were obtained at each time point (pre-treatment and one-month post-treatment), demonstrating consistent patterns; representative values are reported descriptively. A new sensor was used for each recording session.

The system automatically quantified occlusion time (OT), disocclusion time (DT), force distribution per quadrant, and center of force (COF) trajectory.

Measurements were performed before and one month after orthodontic treatment using T-Scan Novus v.9.

### 2.4. Results

Digital occlusal analysis was performed using the T-Scan Novus system before orthodontic treatment with clear aligners and one month after completion of active therapy. At each point, occlusion time (OT), disocclusion time (DT), and center of force (COF) trajectory were recorded and analyzed.

#### 2.4.1. Occlusal Force Distribution

**Figure 5:**
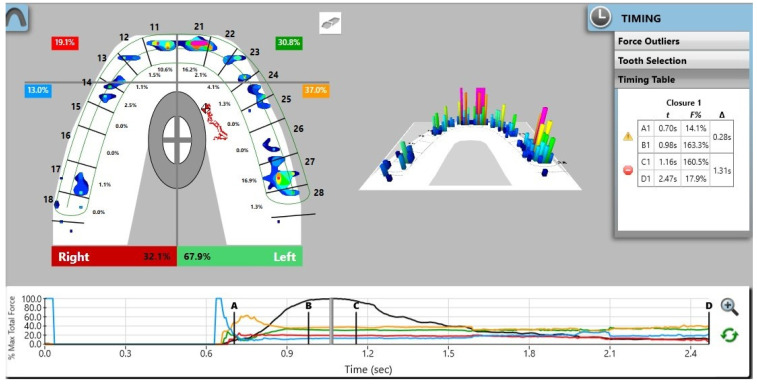
Digital occlusal measurements recorded using the T-Scan Novus system before orthodontic treatment.

**Figure 6:**
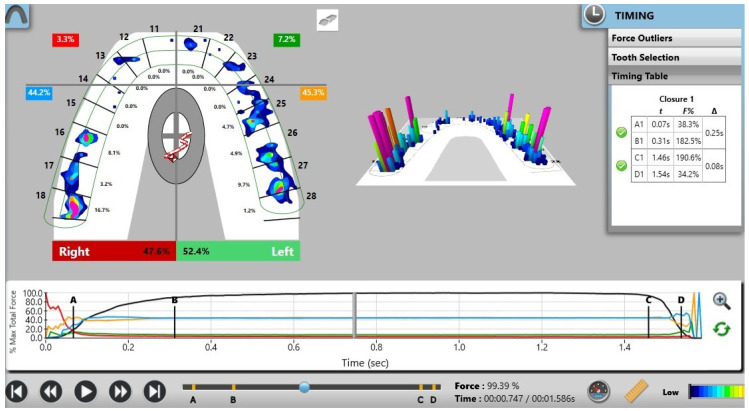
Digital occlusal measurements recorded using the T-Scan Novus system after orthodontic treatment.

Pre-treatment recordings demonstrated a marked asymmetry in occlusal force distribution, with a predominance on the left side (67.9%) compared with the right side (32.1%) (Figure 5). Post-treatment analysis revealed a more balanced bilateral force distribution, with approximately 52.4% of the total occlusal force on the left side and 47.6% on the right side (Figure 6). These findings indicate a reduction in left–right force imbalance compared with baseline. The percentage distribution of occlusal forces before and after treatment is summarized in Table 1.

#### 2.4.2. Occlusion and Disocclusion Timing

Before treatment, prolonged occlusal contact timing was observed, as reflected by increased occlusion time (OT) and disocclusion time (DT) values. Following aligner therapy, a reduction in both parameters was recorded (OT = 0.25 s; DT = 0.08 s) (Table 2). Although these values suggest an improvement in contact timing relative to the pre-treatment condition, they represent measurements from a single patient at a single post-treatment time point and should therefore be interpreted descriptively rather than as evidence of definitive functional normalization.

#### 2.4.3. Center of Force (COF) Trajectory

Analysis of the center of force trajectory demonstrated a more centralized and stable path during maximum intercuspation in the post-treatment recordings compared with the pre-treatment condition. The COF trajectory remained within the physiologic target zone throughout the closing phase, indicating a more symmetrical distribution of occlusal contacts at peak intercuspation.

#### 2.4.4. Qualitative Interpretation

No visually apparent premature contacts were identified in the post-treatment T-Scan recordings. This observation is based on digital occlusal mapping and should be considered a supportive finding rather than a standalone diagnostic criterion.

**Figure 7:**
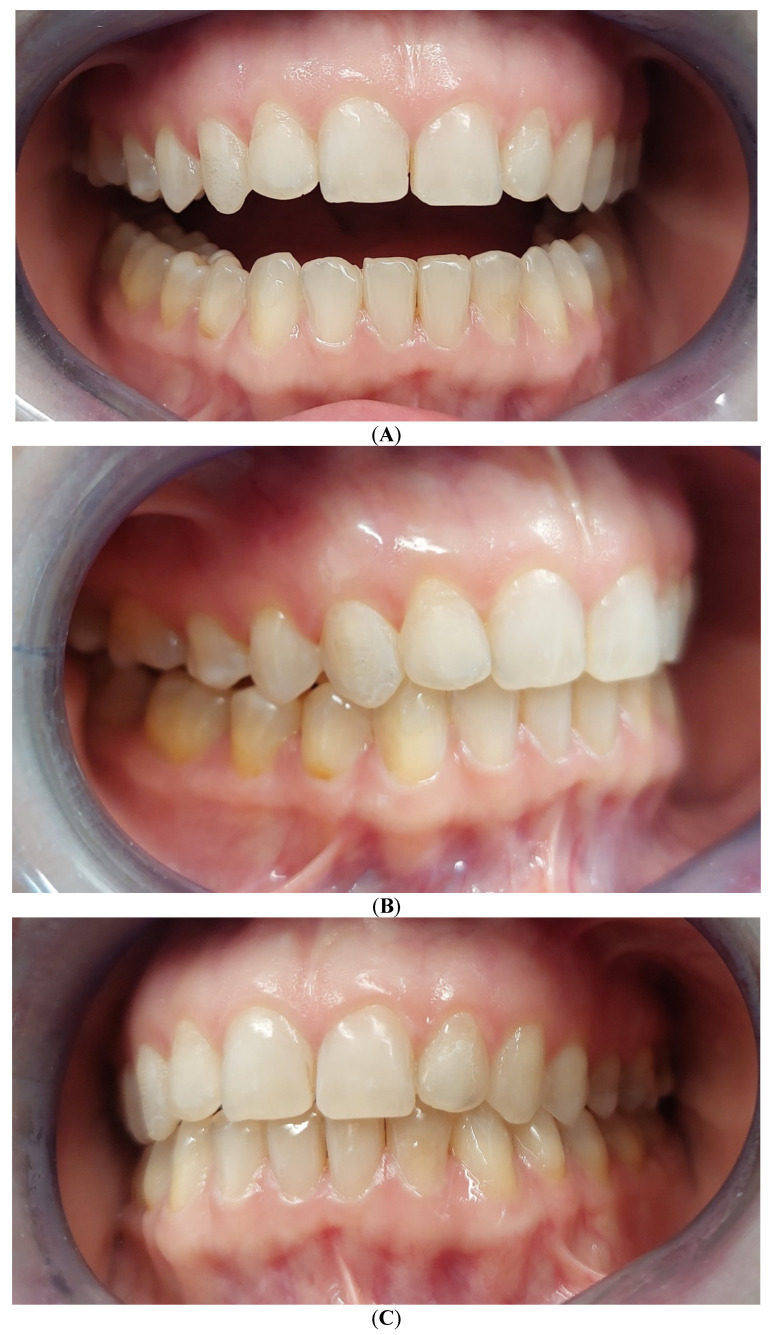
(**A**–**C**) Occlusion 1 month after treatment.

Figure 7 illustrates the occlusal condition recorded one month after completion of active aligner therapy. In addition, follow-up clinical examinations and digital occlusal assessments were scheduled and performed at three and six months after treatment to monitor occlusal adaptation and short-term stability.

## 3. Discussion

This clinical case illustrates the application of digital occlusal analysis for the descriptive assessment of occlusal force distribution and contact timing following orthodontic treatment with clear aligners. The T-Scan Novus system allows real-time visualization of occlusal contact sequences and relative force distribution, providing objective, dynamic information that complements conventional qualitative occlusal indicators such as articulating paper [1,2].

Before treatment, the patient demonstrated pronounced asymmetry in occlusal force distribution, with a clear predominance on one side. Post-treatment recordings showed a shift toward a more balanced bilateral force distribution. Similar trends have been described in cross-sectional orthodontic studies using T-Scan III and Novus systems; however, such population-level observations cannot be directly extrapolated to individual clinical outcomes [5].

Occlusion time (OT) reflects the interval between initial tooth contact and maximum intercuspation, whereas disocclusion time (DT) represents the transition from full occlusal contact to anterior guidance. Although reference values for these parameters have been proposed in the literature, reported thresholds vary depending on methodology, sensor calibration, and patient-specific factors [4,6]. In the present case, a reduction in DT was observed after treatment, while OT increased at the one-month follow-up. These findings likely reflect short-term occlusal adaptation rather than definitive functional stabilization, particularly given the limited follow-up period.

The visualization of force distribution patterns and timing parameters may facilitate the identification of occlusal imbalances that are not readily detectable with static clinical methods alone. Previous studies suggested that digital occlusal analysis may be useful during splint therapy, prosthetic rehabilitation, orthodontic finishing, and retention phases as a supportive tool for monitoring occlusal changes over time [1,8]. Nevertheless, its output remains technique-sensitive and should be interpreted in conjunction with clinical findings rather than as isolated diagnostic endpoints.

Taken together, this case demonstrates the feasibility of using the T-Scan Novus system to document short-term changes in occlusal parameters following clear aligner therapy. However, given the single-patient design, absence of repeated measurements, and short follow-up, the findings should be regarded as illustrative. Larger prospective studies with standardized recording protocols and longer observation periods are required to clarify the clinical relevance and reproducibility of digital occlusal metrics in orthodontic outcome assessment [2,4].

### 3.1. Limitations of the Study

The primary limitation of this report is its single-case design, which precludes generalization of the findings. Additionally, the short post-treatment follow-up period does not allow conclusions to be made regarding long-term occlusal stability. Measurement variability inherent to digital occlusal analysis and the absence of patient-reported functional outcomes should also be acknowledged.

### 3.2. Recommendations for Future Research

Future studies should include larger patient cohorts, longer follow-up periods, and repeated measurements to assess occlusal stability over time. The integration of patient-reported outcomes and complementary functional assessment tools may further enhance the clinical relevance of digital occlusal analysis in orthodontic and prosthetic workflows.

## 4. Conclusions

In the present clinical case, digital occlusal analysis using the T-Scan Novus system enabled an objective assessment of occlusal force distribution and contact timing before and after clear aligner therapy. The results indicated an improvement toward more balanced occlusal loading and shorter contact timing, supporting short-term functional occlusal stability. Although these findings underscore the clinical usefulness of T-Scan Novus analysis, further studies involving larger patient samples and longer follow-up are necessary to support its routine integration into orthodontic and prosthetic treatment protocols.

## Figures and Tables

**Table 1:** Percentage of occlusal force before and after treatment.

	Occlusal Force %			
	Right Anterior Quadrant (RAQ)	Left Anterior Quadrant (LAQ)	Right Posterior Quadrant (RPQ)	Left Posterior Quadrant (LPQ)
**B** **efore treatment**	19.1%	30.8%	13%	37%
**After treatment**	3.3%	7.2%	44. 2%	45.3%

**Table 2:** OT and DT values before and after treatment.

	Occlusion Time (OT)	Disocclusion Time (DT)
**Before treatment**	0.28 s	0.25 s
**After treatment**	1.31 s	0.08 s

## Data Availability

The research data are available upon request by contacting the corresponding author. The data is not publicly available due to privacy concerns.
